# A Soft Mechanoluminescent Skin for High‐Resolution Optical Tactile Sensing in Human–Machine Interaction

**DOI:** 10.1002/advs.75507

**Published:** 2026-05-05

**Authors:** Yu Feng, Qiaojiao Wang, Yehui Liu, Jiankun Li, Senlin Hou, Xiaomeng Yang, Ziyi Li, Yanji Yi, Meng Chen, Guanglie Zhang, Hao Sun, Wen Jung Li

**Affiliations:** ^1^ Department of Mechanical Engineering City University of Hong Kong Hong Kong SAR China; ^2^ CAS‐CityU Joint Laboratory for Robotic Research City University of Hong Kong Hong Kong SAR China; ^3^ Department of Precision Machinery and Precision Instruments University of Science and Technology of China Hefei China; ^4^ School of Mechatronics Engineering Harbin Institute of Technology Harbin China; ^5^ State Key Laboratory of Robotics and Systems Harbin Institute of Technology Harbin China

**Keywords:** human–machine interaction, mechanoluminescence, soft sensor, tactile sensor

## Abstract

As soft interfaces become central to robotics, wearables, and human–machine interaction, a persistent challenge is to sense touch with high fidelity while keeping devices simple, robust, and negligible power requirement at the sensitive element. Herein, we report a soft mechanoluminescent (ML) tactile sensor converting force directly into light for imaging‐based readout, integrating a thin, three‐layer ML‐skin with a CMOS module. Under mechanical stimulation, BaTiO_3_ inclusions intensify local piezoelectric fields to excite ZnS:Cu emitters, producing light without electrical bias, pixel wiring, or external illumination. This optical transduction provides intrinsic electrical isolation while enabling scalable, high‐density spatial mapping, where resolution is defined by optics rather than electrode routing. Coupled to a 640 × 480, 30 Hz CMOS array, the ML‐sensor achieves a sensitivity of 27.5 N^−1^, a 30 ms response time, ∼80 µm spatial resolution, and stable operation for over 8000 cycles. Furthermore, ML‐sensor enables real‐time handwriting recognition and human–machine interaction, demonstrating its potential as a natural tactile interface. By merging force‐to‐light conversion with a minimal device stack and vision‐native readout, this work outlines a pathway to energy‐efficient, conformal touch interfaces scalable across next‐generation soft electronics and interactive systems.

## Graphical abstract

A soft mechanoluminescent tactile sensor that converts force directly into light is presented, enabling imaging‐based, wiring‐free touch sensing. By integrating a flexible ML‐skin with CMOS readout, the system achieves high sensitivity, fast response, and high spatial resolution, while maintaining structural simplicity and energy efficiency, offering a scalable pathway for next‐generation human–machine interfaces.

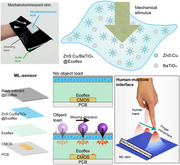

## 1 Introduction

Soft tactile sensors have become essential components in intelligent robotics [1, 2, 3, 4], wearable electronics [5, 6, 7], and human–machine interfaces [8, 9, 10]. As the demand for real‐time perception and efficient interaction continues to rise, these sensors are expected to simultaneously offer high sensitivity, high spatial resolution, rapid response, and desired mechanical compliance. Additionally, they must feature simplified device architectures and enable direct, efficient signal readout to minimize overall power consumption and system complexity. A variety of soft tactile sensors have been developed, such as capacitive [11, 12, 13, 14, 15], resistive [16, 17], piezoelectric [18, 19, 20, 21], ion‐conductive [22, 23, 24], and optoelectronic sensors [25, 26, 27, 28, 29]. These sensors offer benefits in terms of sensing performance, flexibility, and adaptability to various applications. However, electrical tactile sensors rely on multi‐electrode arrays and complex wiring, which increase structural and packaging complexity, making them susceptible to signal crosstalk and noise, and thereby limiting their spatiotemporal resolution and reliability [30, 31, 32]. Moreover, continuous power supply and multi‐channel signal acquisition result in high energy consumption [33, 34]. On the other hand, although optoelectronic tactile sensors exhibit strong immunity to electromagnetic interference, they typically require external light sources, reflective structures, or bulky optical components, making it difficult to achieve conformal flexibility, structural simplicity, and high energy efficiency in practical applications [35, 36].

In recent years, mechanoluminescent materials have offered new opportunities for developing high‐efficiency tactile sensors owing to their unique force‐induced light‐emission characteristics [37, 38, 39, 40]. These materials generate optical signals in response to mechanical stimulation, enabling the passive conversion of mechanical energy into optical energy [41, 42, 43, 44, 45]. Unlike conventional capacitive or resistive tactile arrays that rely on distributed electrode networks and multiplexed electrical readout, the proposed mechanoluminescent sensing approach shifts the signal transduction from the electrical to the optical domain. This architecture eliminates pixel‐level wiring and enables parallel signal acquisition via imaging, thereby reducing system complexity and mitigating electrical crosstalk. The emission location and intensity intrinsically carry spatiotemporal information. As a result, mechanoluminescent tactile sensing can be achieved without the need for electrode wiring or external illumination, and without electrical bias at the sensing layer. Compared with electrical and optoelectronic tactile sensors, mechanoluminescent systems offer advantages in structural simplicity, immunity to electromagnetic interference, and energy utilization efficiency [46, 47, 48, 49, 50, 51, 52]. However, existing mechanoluminescent tactile sensors still suffer from limited emission intensity, insufficient mechanical flexibility, and low integration compatibility, which restrict their applicability in practical tactile sensing scenarios.

**FIGURE 1:**
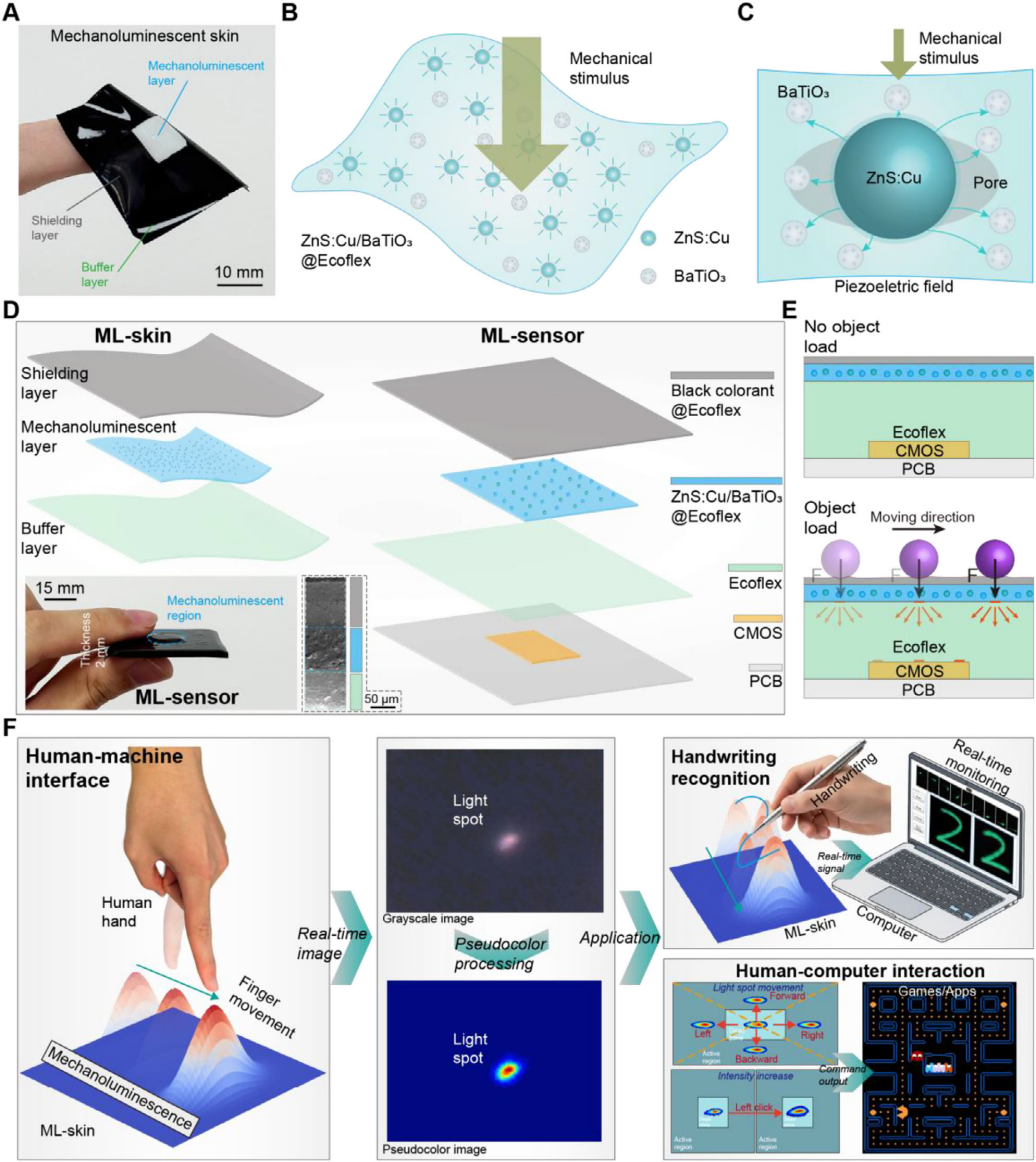
Working principle and design of the mechanoluminescent tactile sensor (ML‐sensor). (A) Optical photograph of the flexible ML‐skin. (B) Schematic illustration of the mechanoluminescence generated from the ML‐skin under mechanical stimulation. (C) Mechanistic diagram showing the light‐emission process of ZnS:Cu and the polarization‐enhanced local electric field contributed by BaTiO_3_ nanoparticles embedded in the Ecoflex matrix. (D) Structural design and photograph of the integrated ML‐sensor. (E) Working principle of the ML‐sensor, in which mechanical deformation induces mechanoluminescence for tactile signal transduction. (F) Demonstration of ML‐sensor applications as a human–machine interface, including real‐time handwriting recognition and interactive control based on mechanoluminescence imaging.

Here, we present a soft mechanoluminescent tactile sensor (ML‐sensor) composed of an ML‐skin and a CMOS imaging chip. As shown in Figure 1A, the ML‐skin (∼250 µm thickness) consists of a shielding layer, a mechanoluminescent layer, and a buffer layer, and is capable of generating stable light signals under mechanical stimulation. It enables the optical transduction of mechanical inputs without the need for wiring, external illumination, or electrical bias at the sensing layer, and cooperates with CMOS to form a structurally simple and efficient tactile sensing system (see Table S1 for comparison). Furthermore, the local piezoelectric field generated by BaTiO_3_ under mechanical loading promotes carrier separation and recombination in ZnS:Cu, substantially enhancing the mechanoluminescent intensity and enabling efficient force‐to‐light conversion. Leveraging the high spatial sampling capability of the CMOS array (640 × 480, 30 Hz) and the enhanced emission performance of the ZnS:Cu/BaTiO_3_@Ecoflex composite, the ML‐sensor exhibits desired sensing performance (specifications comparison in Table S2), i.e., high sensitivity (27.5 N^−^^1^), millisecond‐level response speed (30 ms), high spatial resolution (80 µm^2^), and reliable repeatability (over 8000 cycles). We further demonstrate real‐time handwriting trajectory recognition and real‐time human–machine interaction control, confirming its potential as an efficient tactile interface. Overall, this work realizes a soft tactile sensor through an efficient force‐to‐light conversion mechanism and a simplified device structure, offering a new pathway toward high‐performance flexible tactile interfaces.

## 2 Results and Discussion

### 2.1 Principle and Design of ML‐Sensor

Figure 1B illustrates the mechanoluminescent layer of the ML‐sensor, in which ZnS:Cu and BaTiO_3_ particles are uniformly embedded within the Ecoflex elastomer. When an external mechanical stimulus is applied, the elastic matrix deforms and transfers stress to the dispersed ZnS:Cu particles. This process activates the trap states within ZnS:Cu and leads to the release of photons, producing a visible mechanoluminescent signal [53, 54, 55]. The presence of BaTiO_3_ further enhances this process [56, 57, 58]. Mechanical deformation of BaTiO_3_ generates a localized piezoelectric field, as depicted in Figure 1C, which facilitates charge separation, accelerates carrier migration, and increases the probability of radiative recombination within the ZnS:Cu particles. The combined effect of stress‐induced activation in ZnS:Cu and field‐assisted luminescence promoted by BaTiO_3_ results in a stronger and more responsive optical output. This synergistic mechanism forms the foundation of the ML‐sensor and enables the real‐time conversion of mechanical inputs into robust optical signals.

Based on this mechanism, we designed a structurally simple ML‐skin, as illustrated in Figure 1D. The ML‐skin is a flexible multilayer membrane composed of three Ecoflex‐based layers arranged from top to bottom (Figure S1 and Movie S1). It is worth noting that the Ecoflex‐based composite used in the mechanoluminescent layer maintains desired mechanical flexibility, as evidenced by its high elongation at break and robust tensile performance (Figure S4), which ensures reliable operation under repeated deformation. These layers include a shielding layer made of black‐colorant‐doped Ecoflex, a mechanoluminescent layer consisting of ZnS:Cu/BaTiO_3_@Ecoflex, and a buffer layer formed by pristine Ecoflex. The total thickness of the membrane is ∼250 µm. By laminating and conformally attaching the ML‐skin onto a CMOS module, an integrated ML‐sensor can be readily fabricated. Within the ML‐sensor architecture, the bottom buffer layer protects the CMOS module from direct mechanical impact during external stimulation. Its optical transparency ensures that the emitted mechanoluminescent light can reach the CMOS chip with minimal attenuation. The top shielding layer blocks ambient light and allows the CMOS to detect only the emission generated by the mechanoluminescent layer. This configuration effectively suppresses noise and significantly enhances the overall sensing quality.

The force‐sensing mechanism of the ML‐sensor is illustrated in Figure 1E. In the absence of external pressure, the mechanoluminescent layer remains inactive and no optical emission is generated, resulting in no detectable response from the CMOS module. When an external force is applied, the mechanoluminescent layer is mechanically stimulated and emits light, and the CMOS then records the spatiotemporal optical signals across the entire sensing area of 6.5 × 4.3 mm (1 × 1 pixel = 80 µm^2^). Through this mechanoluminescent process, a direct relationship is established between the applied force and the corresponding optical output, enabling the ML‐sensor to perform force‐based tactile sensing. This operating principle provides the fundamental basis for employing the ML‐sensor as an interactive interface in human–computer interaction tasks.

### 2.2 Fabrication and Characterization of ML‐Skin

**FIGURE 2:**
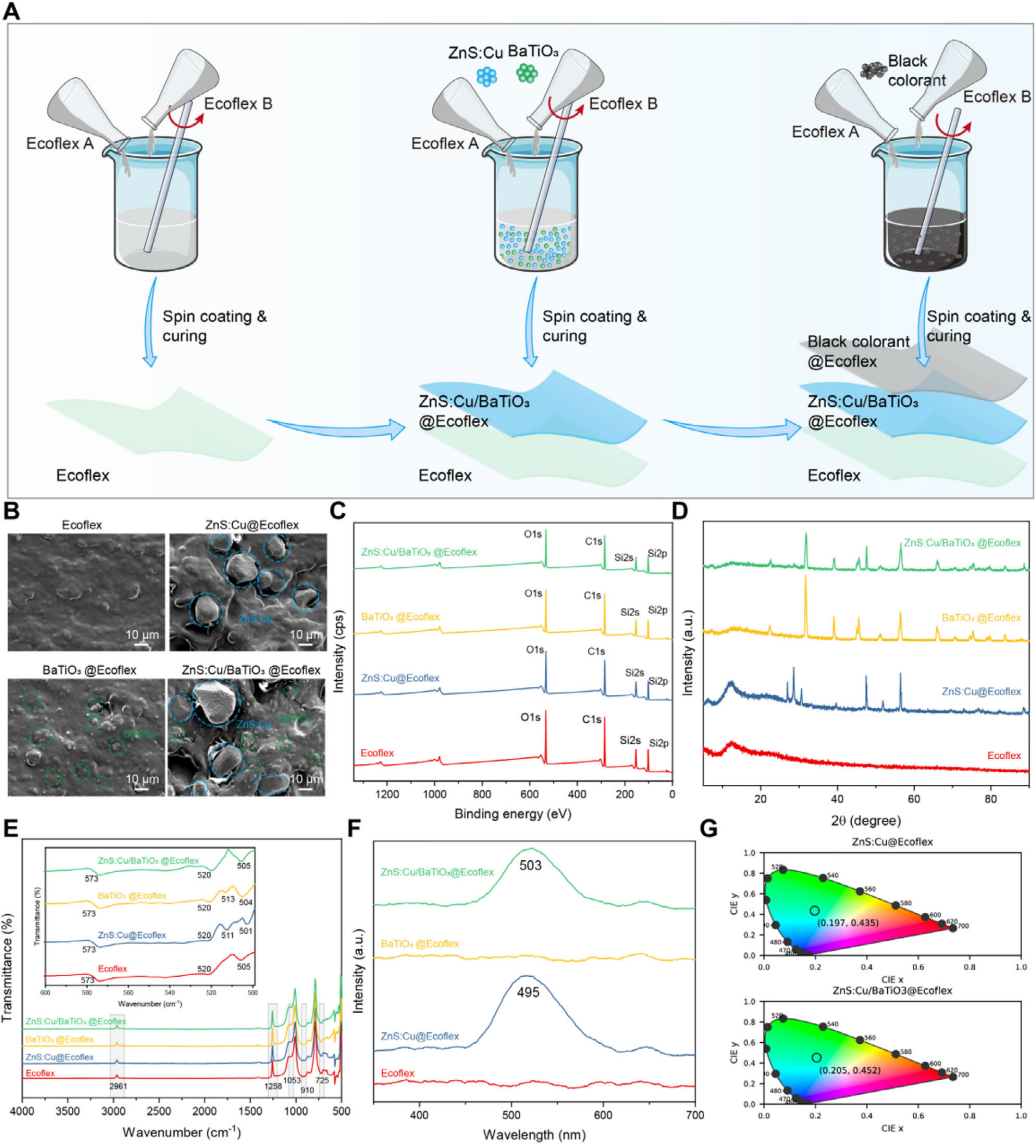
Fabrication and characterization of the ML‐skin. (A) Schematic illustration of the fabrication process of the ML‐skin. (B) SEM images of the ML‐skin composite. (C) XPS spectra of the prepared elastic composites (Ecoflex, ZnS:Cu@Ecoflex, BaTiO_3_@Ecoflex, and ZnS:Cu/BaTiO_3_@Ecoflex). (D) XRD patterns of the corresponding elastic composites. (E) FTIR spectra of the prepared elastic composites. (F) Photoluminescence spectra of the prepared elastic composites. (G) CIE chromaticity diagram of the mechanoluminescent emission from ZnS:Cu@Ecoflex and ZnS:Cu/BaTiO_3_@Ecoflex.

The ML‐skin was fabricated through a classical layer‐by‐layer spin‐coating process, as illustrated in Figure 2A. The procedure consisted of three sequential coating and curing steps. First, Ecoflex Part A and Part B were mixed thoroughly at a 1:1 mass ratio and spin‐coated to form a ∼60 µm buffer layer, followed by curing at 80°C for 5 h. Subsequently, ZnS:Cu and BaTiO_3_ powders were incorporated into uncured Ecoflex to form a homogeneous composite slurry, which was spin‐coated onto the pre‐cured Ecoflex layer to obtain a ∼90 µm mechanoluminescent layer. This layer was cured at 70°C for 6 h. Finally, a black‐colorant‐modified Ecoflex precursor was spin‐coated onto the bilayer film to form a ∼100 µm optical shielding layer and cured at 70°C for another 6 h. The trilayer film was then cut into sheets with dimensions of 45 mm × 45 mm × 0.25 mm to obtain the ML‐skin. Due to this sequential spin‐coating and curing process using the same elastomeric matrix, strong interfacial bonding is formed between adjacent layers through interpenetration and crosslinking continuity, ensuring structural integrity under repeated deformation. Owing to its simple and scalable fabrication process, this approach is advantageous for large‐area production. When laminated onto a CMOS module through natural electrostatic adhesion, the ML‐skin forms the complete ML‐sensor (Figure S5). This electrostatic adhesion provides sufficient contact stability during mechanical interactions, ensuring consistent optical coupling between the ML‐skin and the CMOS sensor (Movie S5).

The microstructural characteristics of the ML‐skin were first examined using a scanning electron microscope (SEM) and elemental mapping (Figure S3), as shown in Figure 2B. Pristine Ecoflex exhibited a smooth and uniform elastomeric matrix, whereas ZnS:Cu@Ecoflex and BaTiO_3_@Ecoflex displayed uniformly distributed ZnS:Cu and BaTiO_3_ particles embedded within the polymer network. In the ZnS:Cu/BaTiO_3_@Ecoflex composite, both functional fillers were co‐dispersed without noticeable agglomeration, indicating strong interfacial compatibility and good structural integrity of the mechanoluminescent layer, which is essential for achieving efficient coupling between the mechanical input and the optical emission. X‐ray photoelectron spectroscopy (XPS) survey spectra (Figure 2C; Figure S2) of all films revealed characteristic C1s, O1s, and Si2s/Si2p peaks originating from the Ecoflex matrix, with no additional peaks arising from ZnS:Cu or BaTiO_3_. This confirms that incorporation of the fillers does not alter the surface chemical environment of the elastomer, ensuring chemical stability of the active layer during mechanoluminescent excitation.

X‐ray Diffraction (XRD) patterns (Figure 2D) further demonstrated that the crystalline structures of ZnS:Cu and BaTiO_3_ were retained within the composites, with clear diffraction peaks observed in the corresponding films, whereas pristine Ecoflex exhibited only a broad amorphous halo. The preserved crystallinity of the functional nanoparticles is crucial for achieving stable luminescent performance and for enhancing local electric fields under mechanical stimulation. In addition, Fourier transform infrared spectroscopy (FTIR) spectra (Figure 2E) of all samples exhibited signature Si─O─Si, Si─CH_3_, and Si─H vibrational bands characteristic of Ecoflex, without the appearance of new absorption peaks. This indicates that no significant chemical reactions occur between the fillers and polymer matrix, and that the crosslinked silicone network remains intact, maintaining the flexibility and structural robustness of the ML‐skin.

Furthermore, photoluminescence measurements (Figure 2F) revealed that ZnS:Cu@Ecoflex exhibited a characteristic emission peak at ∼495 nm originating from Cu^+^ activator centers. Upon incorporation of BaTiO_3_, the emission peak of ZnS:Cu/BaTiO_3_@Ecoflex slightly red‐shifted to ∼503 nm and showed substantially enhanced intensity, suggesting that the local electric field generated by BaTiO_3_ facilitates carrier excitation and radiative recombination. Pure Ecoflex showed no detectable emission. The CIE chromaticity shown in Figure 2G further supports the photoluminescence trends. ZnS:Cu@Ecoflex emits at (0.197, 0.435), and ZnS:Cu/BaTiO_3_@Ecoflex exhibits a slight shift to (0.205, 0.452), which agrees well with its enhanced emission intensity. These results confirm that the ML‐skin maintains stable chromatic properties, while BaTiO_3_ plays a beneficial role in improving emission efficiency without compromising color stability.

### 2.3 Sensing Performance of ML‐Sensor

The sensing performance of the ML‐sensor, which operates based on the mechanoluminescent mechanism. As shown in Figure S6, the sensing performance of the ML‐sensor, which operates based on the mechanoluminescent mechanism, was evaluated using a real‐time optical acquisition pipeline. The mechanoluminescent signals generated by external forces were captured by the CMOS module and processed through a dedicated algorithm that performs noise filtering, background subtraction, adaptive thresholding, and feature extraction (typical images in Figure S7 and Movie S2). It is worth noting that the ML‐skin also demonstrates a clear stretch‐induced mechanoluminescence (Movie S6). To optimize the composition of the mechanoluminescent layer, ML‐skins with different ZnS:Cu/BaTiO_3_ formulations were evaluated. Figure S8A presents photographs of the corresponding ML‐skins with ZnS:Cu:BaTiO_3_ ratios of 1:0, 1:1, 2:1, 3:1, and 4:1. As shown in Figure S8B, the 1:0 and 1:1 formulations exhibited relatively weak ML responses, with average intensities of 83.4 and 101.8 at 5 N, respectively, and also showed pronounced nonlinearity (three‐stage linear fitting). This may be attributed to the limited piezoelectric‐assisted enhancement in the absence of, or at low levels of, BaTiO_3_, which results in a relatively low mechanoluminescent output. As the ZnS:Cu/BaTiO_3_ ratio was further increased, although the 3:1 and 4:1 formulations exhibited higher sensitivities than the 2:1 formulation (maximum sensitivities of 42.4 and 40.9 N^−1^, respectively), their ML performance began to decline beyond the 2:1 ratio (104.5, 93.1, and 95, respectively), while nonlinearity still remained. The 2:1 formulation provided the optimum balance among ML output, sensitivity, and linearity, and was therefore selected for all subsequent sensing measurements. In addition, the minimum detectable force was examined for the different formulations (Figure S8C). As the applied force increased from 0 to 0.2 N (step 0.05 N), the introduction of BaTiO_3_ was found to slightly increase the minimum detectable force of the ML‐skin from 0.1 N to 0.15 N. This may be attributed to the combined effects of local electric‐field enhancement, effective ZnS:Cu content, and stress transfer within the composite layer under low‐force loading.

**FIGURE 3:**
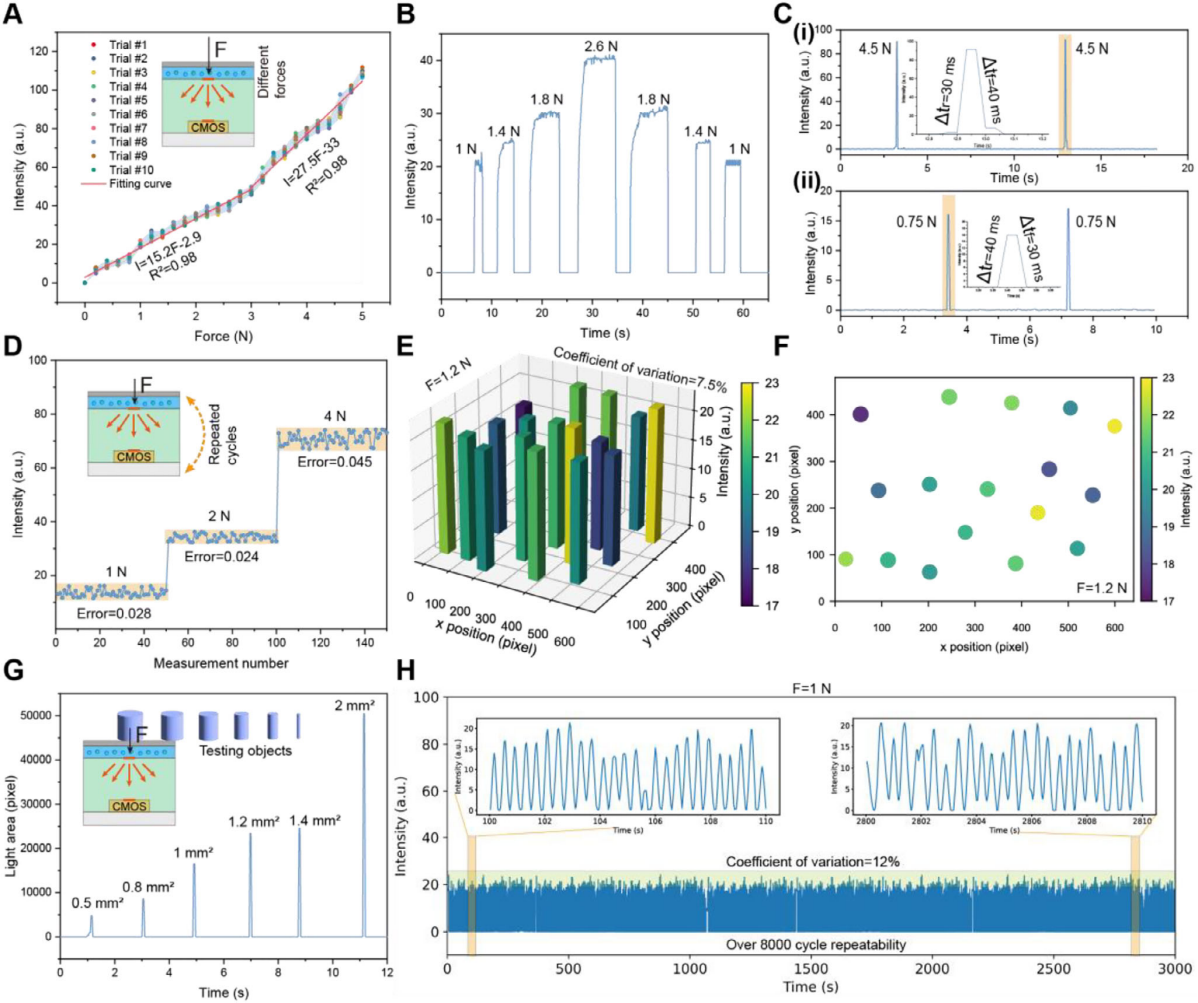
Sensing performance of the ML‐sensor. (A) Relationship between the ML‐sensor's mechanoluminescent intensity and applied force. (B) Dynamic intensity response of the ML‐sensor under varying force levels. (C) Time‐resolved intensity response of the ML‐sensor during force loading. (D) Intensity response of the ML‐sensor during repeated loading and unloading cycles at 1 N, 2 N, and 4 N. (E) Intensity response of the ML‐sensor when forces were applied at different spatial positions. (F) Consistency analysis of the ML‐sensor's spatial intensity mapping. (G) Intensity response of the ML‐sensor when detecting objects with different contact areas (0.5, 0.8, 1.0, 1.2, 1.4, and 2.0 mm^2^). (H) Repeatability of the ML‐sensor over 8000 cyclic force loadings.

As shown in Figure 3A, the mechanoluminescent intensity increases steadily with applied force over the 0–5 N range, rising from 0 to ∼120. Two approximately linear regimes can be empirically fitted, with a sensitivity of 15.2 N^−1^ in the 0–3 N region and 27.5 N^−1^ in the 3–5 N region, and both regions exhibit linearity with an R^2^ value of 0.98. Ten repeated measurements closely follow the fitted curves, confirming high quantitative stability across the entire force range. Dynamic force loading further highlights the sensor's capability, as shown in Figure 3B. Stepwise increases in force (1, 1.4, 1.8, and 2.6 N) yield corresponding intensities of ∼20, ∼28, ∼33, and ∼42, representing a total amplitude change of ∼110%. The sharp rising and falling edges during each loading event demonstrate rapid response and recovery, while the nearly identical peak intensities under repeated loading at the same force indicate excellent dynamic repeatability.

Furthermore, temporal response characteristics were investigated under both high and low forces (Figure 3C). The ML‐sensor exhibits an ultrafast response, with rise and decay times of ∼30 and ∼40 ms at 4.5 N, respectively. Remarkably, these values remain unchanged at a lower force of 0.75 N, indicating that the sensor's intrinsic response speed is largely independent of the applied load. The narrow pulse profiles confirm the high temporal resolution of the ML‐sensor, making it suitable for real‐time tactile sensing and rapid human–machine interaction scenarios. Repeated loading and unloading cycles (Figure 3D) further demonstrate the outstanding operational stability of this system. Steady‐state intensities of ∼20, ∼35, and ∼75 are observed at 1, 2, and 4 N, respectively, consistent with the force‐dependent sensitivity. Across more than 150 consecutive measurements, the relative errors remain low (0.028, 0.024, and 0.045), with negligible drift, underscoring the sensor's reliability under cyclic mechanical stimuli.

Spatial performance was further examined by applying force (1.2 N) at different positions on the ML‐sensor (Figure 3E). The response intensities remain within a narrow range of ∼17–23 despite spatial variations of ∼600 pixels along the *x*‐axis and ∼400 pixels along the *y*‐axis, indicating a spatial consistency (coefficient of variation 7.5%) in the mechanoluminescent layer. Moreover, a broader spatial mapping presented in Figure 3F confirms that the sensor operates in a position‐independent manner. Across a 600 × 450‐pixel area, most responses remain concentrated between 18 and 20, and the overall spatial variation is only about 28%. These results validate the uniformity and reproducibility of the sensor's spatial output across the entire active region. The ML‐sensor also demonstrates the desired capability in distinguishing contact areas (Figure 3G). The emitted light area increases monotonically from ∼3000 pixels (0.5 mm^2^) to ∼50 000 pixels (2.0 mm^2^), with intermediate values clearly separated at 0.8, 1.0, 1.2, and 1.4 mm^2^. The smallest distinguishable interval, represented by the comparison between 0.5 and 0.8 mm^2^, corresponds to an area difference of 0.3 mm^2^.

Finally, the over 8000‐cycle test (Figure 3H) was conducted to verify the operational repeatability of the ML‐sensor within the experimentally accessible range, rather than to define its ultimate service lifetime. The intensity remains stable within ∼18–24 during ∼3000 s of continuous operation (force 1 N) without observable drift. After thousands of actuations, the waveform, amplitude, and baseline remain nearly unchanged (coefficient of variation 12%), demonstrating desired mechanical durability and stable mechanoluminescent performance suitable for extended tactile monitoring. These results (specifications in Table S3) show that the ML‐sensor possesses high sensitivity, fast response, strong spatial uniformity, robust area‐resolving capability, and remarkable long‐term stability, enabling high‐performance tactile sensing and interactive applications.

### 2.4 Demonstration on Handwriting Recognition

Under external forces, the local stress‐induced luminescence response can not only reflect the magnitude of the load but also accurately indicate the location of the force, thus providing the possibility for real‐time visualized location tracking and dynamic trajectory recording. Based on this characteristic, we have developed a manual trajectory collection and recognition system based on ML‐skin (Figure S9).

**FIGURE 4:**
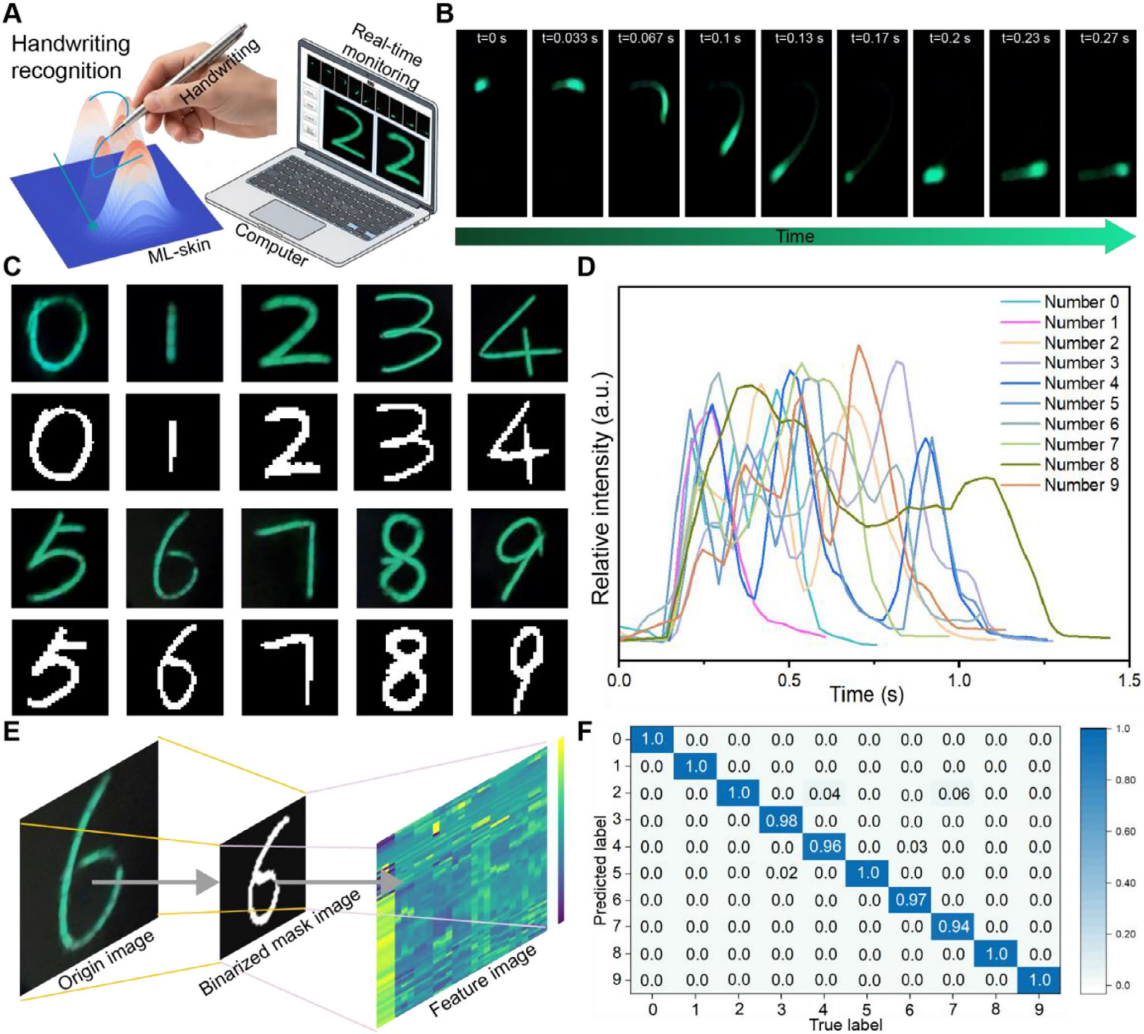
Demonstration of handwriting recognition using the ML‐sensor. (A) Schematic diagram of the image acquisition system for handwriting recognition enabled by the ML‐sensor. (B) Extracted mechanoluminescent images captured during handwriting. (C) Superimposed trajectory images and corresponding binarized mask images of the ML‐sensor when different digits ("0" – "9") were written. (D) Relative intensity response of the ML‐sensor under different handwritten inputs. (E) Schematic workflow of the handwriting recognition process based on mechanoluminescent images collected by the ML‐sensor. (F) Confusion matrix for digit classification using a random forest model based on extracted image features.

Figure 4A illustrates the trajectory collection system, which is composed of ML‐skin, an image acquisition module, and a data processing module. When writing with a 1.2 mm diameter hard pen on the sensor surface, the force applied by the pen tip excites a clear luminescent trajectory in a local area, which is captured in real time by a camera connected to a computer (Movie S3). The resulting video is processed frame by frame to obtain a continuous sequence of single‐frame images, which are then superimposed to reconstruct the complete trajectory. Figure 4B shows segmented images extracted while writing the number “2,” clearly displaying the distribution of the pen stroke trajectory. Figure 4C showcases the writing trajectory images and corresponding Binarized mask images of different numbers (0–9) after image overlay. Figure 4D presents the curve of the average luminescence intensity over time during the writing process, demonstrating the unique spatiotemporal response characteristics of each pen stroke.

In the signal processing and recognition section, as shown in Figure 4E, we initially convert the raw luminescent images into binarized mask images and extract their multidimensional image features for machine learning model training. Based on the extracted image features, we employ the Random Forest algorithm to classify and recognize the handwritten digit samples. The classification results are shown in Figure 4F and Figure S10. The confusion matrix indicates that the system achieves high accuracy in distinguishing the ten numbers (0–9), with a classification accuracy of 98%, highlighting the feasibility and efficiency of combining ML‐sensor trajectory acquisition with machine learning algorithm trajectory recognition. These results demonstrate that the handwriting recognition system integrating mechanical luminescence sensing technology with image recognition algorithms can achieve real‐time imaging of writing trajectories and trajectory recognition. This approach holds great potential in areas such as human–computer interaction based on force‐induced luminescence mechanisms, intelligent sensing, and flexible electronic displays.

### 2.5 Demonstration on Human–Computer Interaction

**FIGURE 5:**
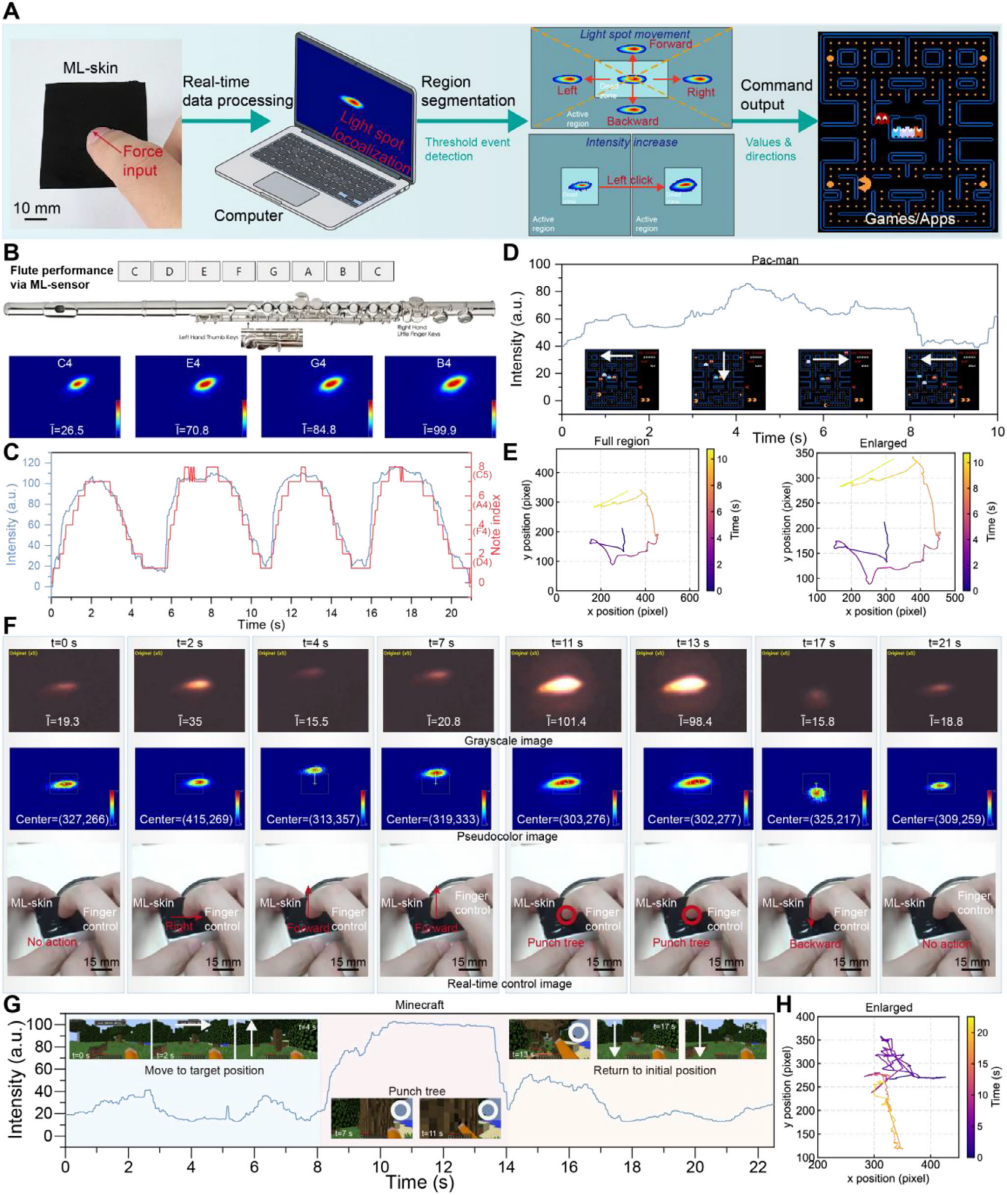
Demonstration of human–computer interaction using the ML‐sensor. (A) Schematic principle of human–computer interaction enabled by using the ML‐sensor as an interactive interface. (B) Pseudocolor images of the ML‐sensor while playing a virtual flute. (C) Intensity response and corresponding note index of the ML‐sensor during the virtual flute performance. (D) Intensity response of the ML‐sensor during Pac‐Man operation. (E) Spatial position response of the ML‐sensor while playing Pac‐Man. (F) Grayscale images, pseudocolor images, and real‐time control images of the ML‐sensor during Minecraft operation. (G) Intensity response of the ML‐sensor while playing Minecraft. (H) Spatial position response of the ML‐sensor during Minecraft gameplay.

The ML‐sensor takes advantage of its high sensitivity and desired spatiotemporal resolution, allowing it to operate as a natural and responsive human–computer interaction interface. Figure 5A presents the interaction mechanism. When a finger presses the ML‐skin, a localized mechanoluminescent spot is produced and captured by the computer in real time. The system processes the acquired images through centroid tracking combined with threshold‐based event detection. The sensing area is divided into functional regions corresponding to forward, backward, leftward, and rightward movements, while a central inactive region helps prevent unintended commands. Once the light spot enters a specific region, the system issues the associated control signal (Movie S4). This process enables smooth interaction with external applications (e.g., virtual flute, Pac‐Man, and Minecraft) and demonstrates that the ML‐skin can function as a reliable vision‐based tactile interface (Figure S11).

To demonstrate the capability of the ML‐sensor as an intuitive human–machine interaction interface, we developed a virtual flute performance demo in which the optical intensity generated by mechanical stimuli is mapped to discrete musical notes. As shown in Figure 5B and Figure S12, different levels of emitted light correspond to specific notes (C4‐C5), enabling the user to play a scale by simply varying the applied pressing force. The real‐time intensity waveform and its corresponding note index are plotted in Figure 5C, clearly illustrating the stable and repeatable optical‐to‐note conversion. This experiment highlights the potential of the ML‐sensor for gesture‐free, force‐modulated musical interaction.

In the Pac‐Man demonstration, the ML‐sensor already enables stable directional control based on the centroid position of the luminescent spot. Figure S13 shows that the centroid moves from (295, 213) to (149, 163), (453, 180), and (322, 338), corresponding to leftward, rightward, and leftward commands. These positional shifts are consistently recognized and translated into real‐time control signals. The intensity response in Figure 4D remains within approximately 40–90, and each actuation produces a clear increase in intensity while the baseline remains steady. Spatial trajectories in Figure 4E span nearly a 300 × 200‐pixel area, forming well‐separated clusters for different commands. The enlarged view indicates that the paths remain distinct over time. These findings demonstrate that the ML‐sensor can accurately resolve continuous motion paths and convert them into stable control inputs, providing a foundation for more advanced interaction tasks.

Building upon the ML‐sensor's positional‐control capability, the Minecraft demonstration integrates an additional action‐triggering mechanism governed by optical intensity, enabling simultaneous navigation and active commands. As shown in Figure 5F, directional control is determined by the centroid displacement of the mechanoluminescent spot, allowing the user to move forward, backward, or sideways within the virtual environment. Meanwhile, the average light intensity serves as the criterion for action execution. During normal navigation, the intensity fluctuates within a low range (typically below ∼35), ensuring that no unintended actions are triggered. When the character approaches the tree, the average intensity increases markedly as the user presses the ML‐skin more strongly, exceeding the action threshold and activating the left‐click command required for punching wood. This transition is clearly reflected in the intensity waveform in Figure 5G, where pronounced peaks correspond to consecutive punching events. After the block is broken, the intensity rapidly returns to baseline levels (< 20), allowing the system to seamlessly revert to navigation‐only mode without false activation. The user then guides the character backward to the starting position, with the intensity remaining low and stable during the retreat. The spatial trajectory plotted in Figure 5H further reveals distinct positional clusters associated with forward motion, punching, and return movement. These clusters occupy a compact spatial window (∼300 × 200 pixels), indicating reliable separation between navigation and action states. Together, these results demonstrate that the ML‐sensor can decode both continuous positional input and discrete intensity‐based events, enabling a compound sequence such as approaching an object, performing an action, and returning to the initial location. These results highlight the ML‐sensor's potential as a natural and intuitive human–machine interaction interface.

## 3 Conclusion

As the demand for tactile interfaces in intelligent robotics, wearable systems, and human–machine interaction continues to grow, the development of soft tactile sensors that offer high performance, low power consumption, and simplified architectures has become increasingly important. In this work, we present a soft mechanoluminescent tactile sensor composed of an ML‐skin and a CMOS imaging chip. The sandwich‐structured ML‐skin and the BaTiO_3_‐assisted enhancement mechanism enable passive and efficient force‐to‐light transduction. The ML‐skin can generate stable mechanoluminescent signals without the need for wiring, external illumination, or electrical bias at the sensing layer. The enhanced emission of the ZnS:Cu/BaTiO_3_@Ecoflex composite and the high spatial sampling capability of the CMOS array (640×480 pixels), the ML‐sensor achieves high sensitivity (27.5 N^−1^), millisecond‐level response speed (30 ms), high spatial resolution (80 µm^2^), and reliable repeatability (over 8000 cycles). The sensor further enables real‐time handwriting trajectory recognition and real‐time human–machine interaction control, demonstrating its potential as a low‐power, efficient, soft tactile interface. While the present study focuses on normal force sensing, it is worth noting that the imaging‐based readout offers the potential to capture spatial deformation patterns associated with more complex loading conditions, such as shear and stretch interactions, which will be explored in future work. Overall, this work establishes a high‐performance soft tactile sensing approach by integrating an efficient force‐to‐light conversion mechanism with a simplified device architecture, offering new material and device strategies for the development of next‐generation energy‐efficient intelligent interfaces and soft electronic systems.

## 4 Experimental Section

### 4.1 Materials

Ecoflex 00–30 (Smooth‐On, Inc., USA) was used as the elastomeric matrix. Zinc sulfide: copper phosphor (ZnS:Cu, product code D502CT) was purchased from Lvmi Technology Co., Ltd. (China). Barium titanate (BaTiO_3_) was obtained from Shanghai Aladdin Biochemical Technology Co., Ltd. (China). The black silicone colorant for the shielding layer was supplied by Puston Silicone Co., Ltd. (China). Unless otherwise specified, all reagents were used as received without further purification.

### 4.2 Preparation of ML‐Skin

The ML‐skin was fabricated using a typical layer‐by‐layer spin‐coating process, as illustrated in Figure 2A. First, Ecoflex Part A and Part B were mixed thoroughly at a 1:1 mass ratio and spin‐coated to form a ∼60 µm thick base film, which was subsequently cured in an oven at 80°C for 5 h. Next, ZnS:Cu and BaTiO_3_ powders were added to uncured Ecoflex and mixed uniformly (mass ratio of Ecoflex:ZnS:Cu:BaTiO_3_ = 5:2:1). The resulting slurry was then spin‐coated onto the pre‐cured Ecoflex base layer to obtain a ∼90 µm thick mechanoluminescent layer, followed by curing at 70°C for 6 h. For the top shielding layer, a black silicone colorant was blended into uncured Ecoflex and spin‐coated onto the bilayer structure to form a ∼100 µm‐thick absorbing layer, which was then cured at 70°C for 6 h. Finally, the trilayer composite film was cut into sheets with dimensions of 45 mm × 45 mm × 0.25 mm to obtain the ML‐skin.

### 4.3 ML‐Sensor Assembly

A USB industrial camera (model 500W_8mm_40 deg, Shenzhen Huarui Viston Technology Co., Ltd., China) was used as the optical detection module. After removing its original lens, the CMOS imaging sensor was mounted onto a custom‐designed holder fabricated via 3D printing (X1 Carbon, Bambu Lab, China). The prepared ML‐skin was then placed directly on top of the exposed CMOS sensor. Owing to the electrostatic attraction between the ML‐skin and the CMOS surface, the film adhered firmly without the need for additional adhesives. This configuration completed the assembly of the ML‐sensor.

### 4.4 Tensile Tests of ML‐Skin

The tensile test was evaluated on a tensimeter (LT‐5000, Reotai Precision Instrument Co., Ltd, China) equipped with a 10 N load cell at a crosshead speed of 30 mm/min. The samples are rectangular in shape, measuring 6 mm in width and 1 mm in thickness, and each sample is tested at least five times.

### 4.5 Sensing Performance Tests of ML‐Sensor

For force‐dependent measurements, the ML‐skin was positioned beneath the indenter of a universal testing machine (ZQ‐990, Zhiqu Precision Instrument Co., Ltd., China). The loading speed was set to 1 mm s^−1^. By applying different normal forces through the testing machine and simultaneously acquiring the corresponding optical responses from the ML‐sensor via the computer, the relationship between the applied force and mechanoluminescent intensity was obtained.

### 4.6 Experimental Setup of ML‐Sensor on Handwriting Recognition

A camera was used to record the luminous trajectory produced when a pen was written on the ML‐skin surface. Each frame of the video was extracted and then superimposed to obtain the complete writing trajectory. The obtained trajectory images were preprocessed for feature extraction, including bounding box width and height normalization, area ratio, 7D moments, 4D distance features, and geometric features (extent, solidity, compactness, eccentricity, orientation, and holes). Subsequently, a random forest machine learning algorithm was used to classify and recognize the writing trajectories of numbers.

### 4.7 Experimental Setup of ML‐Sensor on Human–Computer Interaction

After connecting the ML‐sensor to the computer, an optical position‐threshold control algorithm was employed to process the mechanoluminescent images in real time. The algorithm continuously extracted the centroid position of the emitted light spot and converted its positional changes into corresponding control commands, enabling real‐time human–computer interaction during gameplay.

## Author Contributions

Y.F., G.Z., and W.J.L. conceived the research ideas. Y.F., Q.W., and S.H. prepared and fabricated the materials. Y.F., Q.W., Y.L., and S.H. conducted characterizations. Y.F., Q.W., J.L., and S.H. conducted handwriting recognition experiments. Y.F., Q.W., and Y.L. carried out human–computer interaction experiments. Y.F., Q.W., Y.L., J.L., X.Y., S.H., G.Z., and W.J.L. analyzed the experimental data. Y.F., Q.W., G.Z., and W.J.L. wrote the manuscript. G.Z. and W.J.L. supervised the research. All authors discussed the results and reviewed the manuscript.

## Conflicts of Interest

The authors declare no conflicts of interest.

## Supporting information




**Supporting File 1**: advs75507‐sup‐0001‐SuppMat.pdf.

**Supporting File 2**: advs75507‐sup‐0002‐MovieS1‐S6.zip.

## Data Availability

The data that support the findings of this study are available from the corresponding author upon reasonable request.
