# Supplementary material for: A Soft Mechanoluminescent Skin for High‐Resolution Optical Tactile Sensing in Human–Machine Interaction
Source: Adv Sci (Weinh). 2026 May 5;13(42):e75507. doi: 10.1002/advs.75507 (PMC13335551; doi:10.1002/advs.75507)
Supplement: Supplementary file 1 — Supporting File 1: advs75507‐sup‐0001‐SuppMat.pdf. [file ADVS-13-e75507-s002.pdf]

Supporting Information for

A Soft Mechanoluminescent Skin for High-Resolution Optical Tactile Sensing in Human-Machine Interaction

Yu Feng, Qiaojiao Wang, Yehui Liu, Jiankun Li, Senlin Hou, Xiaomeng Yang, Ziyi Li, Yanji Yi, Meng Chen, Guanglie Zhang, Hao Sun, Wen Jung Li**

Yu Feng, Qiaojiao Wang, Yehui Liu, Jiankun Li, Senlin Hou, Xiaomeng Yang, Ziyi Li, Yanji Yi, Meng Chen, Guanglie Zhang, Wen Jung Li
Department of Mechanical Engineering, City University of Hong Kong, Hong Kong SAR, China

Yu Feng, Jiankun Li, Xiaomeng Yang, Yanji Yi, Meng Chen, Guanglie Zhang, Wen Jung Li
CAS-CityU Joint Laboratory for Robotic Research, City University of Hong Kong, Shenzhen Research Institute, Shenzhen, China

Yanji Yi
Department of Precision Machinery and Precision Instruments, University of Science and Technology of China, Hefei, China

Hao Sun
School of Mechatronics Engineering, Harbin Institute of Technology, Harbin, China

Hao Sun
State Key Laboratory of Robotics and Systems, Harbin Institute of Technology, Harbin, China

Yu Feng, Qiaojiao Wang
Equally contributed authors

E-mail: gl.zhang@cityu.edu.hk, wenjli@cityu.edu.hk

Keywords: mechanoluminescence, tactile sensor, soft sensor, human-machine interaction

Supplementary movie titles

Movie. S1. Flexibility of the ML-skin.

Movie. S2. Real-time mechanoluminescent signal sensing.

Movie. S3. Demonstration on handwriting recognition.

Movie. S4. Demonstration on human-computer interaction.

Movie. S5. Assembly of ML-sensor device.

Movie. S6. Mechanoluminescence by stretching.

Supplementary tables

Table S1. Comparison with state-of-the-art works and this work.

Reference	Sensor Type	Sensing Mechanism	Wiring Required	External Light/Power Needed	Structural Complexity
[1]	Self-powered iontronic capacitive sensor	Variation in the electric double-layer charge distribution under mechanical stimulation	Yes	No	Medium
[2]	Persistent mechanoluminescent composite	Stress-induced release of trapped carriers leading to mechanoluminescent emission	No	No	Medium
[3]	Triboelectroluminescent	Electric-field-induced excitation of ZnS:Cu triggered by triboelectric charge separation	No	No	Medium
[4]	Piezoresistive CNT-PDMS-gel sensor	Change in electrical resistance due to deformation of the CNT conductive network	Yes	Yes	Medium
[5]	Hybrid piezoelectric-triboelectric sensor	Combined piezoelectric polarization and triboelectric charge generation under pressure	Yes	No	High
[6]	Hybrid triboelectric-optoelectronic system	Mechanical-pressure-induced triboelectric output coupled with photodetection-based signal modulation	Yes	Yes	High
[7]	Body-coupled optoelectronic tactile memory device	Electromagnetic body-coupled energy harvesting integrated with deformation-dependent optical response	No	No	High
[8]	Microstructured capacitive sensor	Pressure-induced variation of the dielectric spacing within microtower structures	Yes	Yes	High
[9]	Organic OLED/OPD array	Pressure-dependent redistribution of reflected electroluminescent light detected as photovoltage variation	Yes	Yes	High
**ML-sensor (this work)**	Mechanoluminescent film with CMOS readout	Force-induced mechanoluminescent emission captured as optical intensity variation by a CMOS sensor	No	No	Low

Table S2. Comparison of specifications with state-of-the-art works and this work.

Reference	Spatial Resolution	Response Time	Sensitivity	Durability
[1]	NA	9 ms	1998-24270 pC·N^{-1}	500 cycles
[2]	40000 μm^2	NA	NA	1000 cycles
[3]	NA	NA	NA	12000 cycles
[4]	NA	530 ms	0.14 mV·N^{-1}	NA
[5]	NA	45-85 ms	15.43 / 18.96 V·kPa^{-1}	100000 cycles
[6]	NA	52 ms	0.1875 kPa^{-1}	NA
[7]	NA	NA	NA	NA
[8]	NA	36 ms	1.194 kPa^{-1}	100000 cycles
[9]	202500 μm^2	6 ms	0.11 N^{-1}	NA
**ML-sensor (this work)**	80 μm^2	30 ms	27.5 N^{-1}	Over 8000 cycles

Table S3. Specifications of the ML-sensor.

**Specification**	**Value**
Spatial Resolution	80 μm^2
Response Time	30 ms
Sensitivity	27.5 N^{-1}
Durability	>8000 cycles
Minimum Detectable Force	0.15 N
Size	ML-skin 45 mm×45 mm×0.25 mm
	ML-sensor 45 mm×45 mm×4.5 mm
Weight	6.2 g (ML-skin 0.6 g)

Supplementary texts

Principle of mechanoluminescence

The mechanoluminescent behavior of the ML-skin can be interpreted as a coupled process between stress-driven trap-electron dynamics and piezoelectric-polarization-induced modulation of local electric fields, with its fundamental mechanism originating from the trap architecture and carrier recombination kinetics intrinsic to the ZnS:Cu phosphor system[10]. Under ambient illumination, both shallow and deep traps in ZnS:Cu capture photogenerated electrons and holes. Deep traps, which are typically associated with lattice defects, sulfur vacancies, or Cu-related impurity levels, can retain charge carriers over extended timescales[11]. In the absence of mechanical stimulation, these trapped carriers remain unable to surmount the trap barrier and return to the conduction band, leaving the material in a non-emissive dark state. When external stress acts on the particles and the surrounding polymer matrix, microstructural deformation of the ZnS lattice perturbs the trap energy levels, thereby increasing the probability of electron escape from the traps. The released electrons are promoted to the conduction band and subsequently migrate along the local internal electric field toward Cu^+ luminescent centers, where radiative recombination occurs, and visible photons are emitted, giving rise to the observed mechanoluminescence[12,13].

Within this composite system, the introduced $BaTiO_3$ nanoparticles generate a critical electric-field enhancement effect through piezoelectric polarization. As a representative ferroelectric-piezoelectric material, $BaTiO_3$ develops orientation-dependent polarization when mechanically stressed, producing opposite surface charges on different facets of each particle[14,15]. These polarization charges establish strong localized electric fields in the vicinity of the ZnS:Cu phosphors, effectively lowering the potential barrier for electron de-trapping and enhancing the drift mobility of conduction-band electrons. In addition, previous reports indicate that the

piezoelectric field may induce band bending within ZnS, further promoting electron liberation from traps and substantially increasing the mechanoluminescence intensity[16]. As a consequence, the electro-mechanical coupling between ZnS:Cu and $BaTiO_3$ reduces the excitation threshold, enhances emission brightness, and enables a more sensitive mechanical response.

The soft Ecoflex matrix further contributes to the coupling process by amplifying and transmitting local stress. Owing to its low elastic modulus, externally applied pressure produces pronounced stress concentration around the embedded ZnS:Cu and $BaTiO_3$ particles, leading to microscale compression and relative particle displacement that strengthen trap perturbation and piezoelectric polarization[17,18]. Meanwhile, the viscoelastic characteristics of Ecoflex allow the embedded particles to undergo cyclic microstrain under dynamic loading, which reinforces the carrier capture-release cycle and stabilizes the spatiotemporal characteristics of the mechanoluminescent signal.

CMOS imaging module specifications

A commercial CMOS imaging module (LRCP3205, USB2.0) was used to record the mechanoluminescent signals generated by the ML-skin. The module integrates a 1/2.5-inch optical-format CMOS sensor (5-megapixel class) with an active imaging area of approximately 5.70×4.28 mm^2, corresponding to a native pixel pitch of ~2.2 μm at the maximum resolution of 2592×1944. In this work, the sensor operated at 640×480 resolution and a sampling frequency of 30 Hz, enabling real-time ML readout with stable video acquisition.

Under this operating mode, each digital pixel corresponds to an effective physical sampling interval of ~8.9×8.9 μm^2, equivalent to an imaging area of ~7.9×10^{-5} mm^2 per pixel. This spatial-temporal sampling capability is sufficient to capture the evolution of ML emission patterns during mechanical loading, forming the optical readout foundation of the ML-sensor.

Algorithm of mechanoluminescent signal processing

The algorithm detects optical spot variations by constructing a stable background model and isolating foreground intensity changes. A 30 s sequence is first collected to build a max-value background, which captures the upper bound of ambient illumination and suppresses transient reflections or sensor fluctuations. Each incoming frame is denoised, converted to grayscale, and cropped to a central region of interest to avoid unstable edge artifacts. Background subtraction is then applied to generate a differential intensity map, and negative values are removed to retain only brightness increments associated with true optical activity. This process effectively filters out irrelevant darkening and stabilizes the response under varying environmental lighting. Spot segmentation is achieved through an adaptive dual-threshold strategy combining statistical background noise estimation with a peak-relative threshold derived from the current frame's intensity distribution. This helps eliminate trailing artifacts and spurious highlights. The preliminary binary mask is further refined using morphological opening and median filtering to remove isolated noise pixels and produce a smooth, contiguous spot region. The largest connected component is selected as the valid spot, and its centroid, area, and integrated intensity are computed using image moments for downstream analysis. Together, these steps form a robust, real-time optical spot detection pipeline suitable for sensing, motion tracking, interaction control, and other dynamic measurement scenarios.

Algorithm of human-computer interaction

This human-computer interaction algorithm implements a virtual joystick control system based on an optical tactile sensor. The system first constructs a max-value background model using a 30 s acquisition period, then extracts the light-spot foreground through background subtraction. An adaptive dual-threshold segmentation method, which incorporates noise statistics and peak response characteristics, is applied to suppress motion trails. The centroid of the light spot is computed through image moments, and its displacement relative to a user-defined neutral point is

mapped to directional commands. A proportional deadzone is introduced to prevent jitter, and directional actions are triggered once the displacement exceeds the threshold. Morphological filtering and exponential smoothing further improve robustness, enabling real-time mapping from optical signals to discrete navigation commands.

On top of the positional-control module, the algorithm incorporates an intensity-based action trigger for mouse left-click events. The system computes the average light intensity for each frame and compares it with a predefined threshold. When the average intensity exceeds this threshold, indicating a stronger press, the algorithm registers a left-click action and issues the corresponding command, such as punching a block in Minecraft. This intensity-triggered module operates alongside the centroid-based directional control, allowing the system to execute compound tasks that involve both movement and action initiation while maintaining high stability and responsiveness.

Supplementary figures

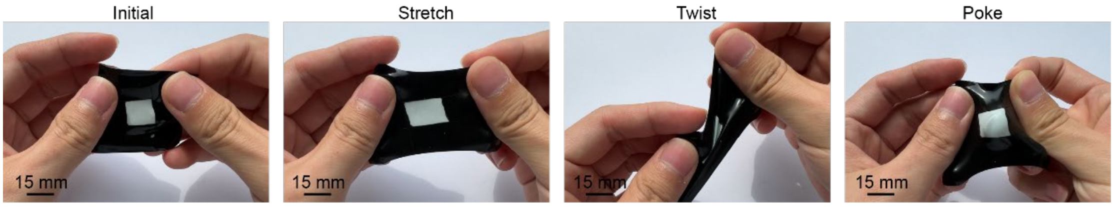

Figure S1. The ML-skin maintains structural integrity under stretching, twisting, and poking deformations.

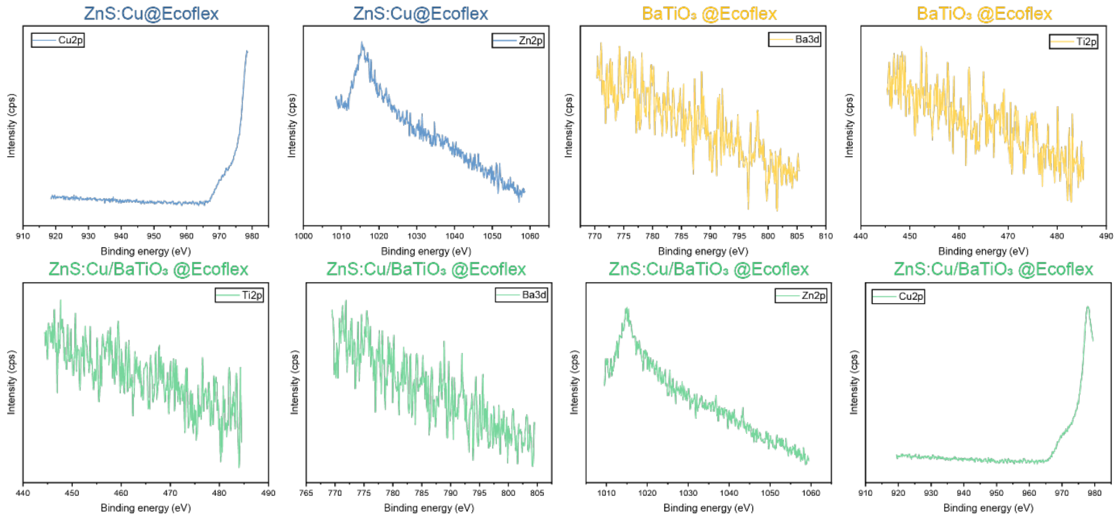

Figure S2. XPS spectra of ZnS:Cu@Ecoflex, $BaTiO_3$@Ecoflex and ZnS:Cu/$BaTiO_3$@Ecoflex composites showing the characteristic Cu2p, Zn2p, Ba3d and Ti2p peaks.

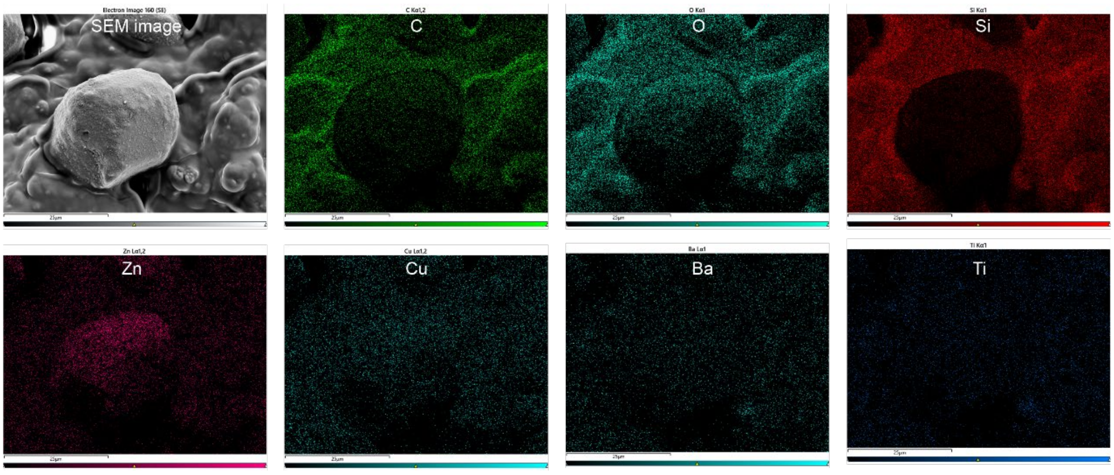

Figure S3. SEM image and corresponding EDS of the ZnS:Cu/$BaTiO_3$@Ecoflex composite, showing the spatial distributions of C, O, Si, Zn, Cu, Ba, and Ti.

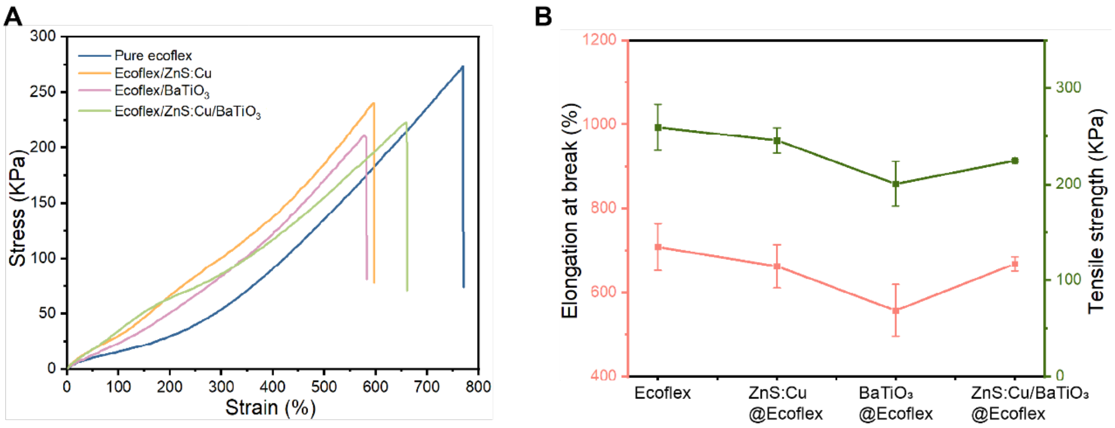

Figure S4. Mechanical properties of Ecoflex-based composites. A) Stress-strain curves of pure Ecoflex, Ecoflex/ZnS:Cu, Ecoflex/$BaTiO_3$ and Ecoflex/ZnS:Cu/$BaTiO_3$. B) Elongation at break and tensile strength of the corresponding composite films.

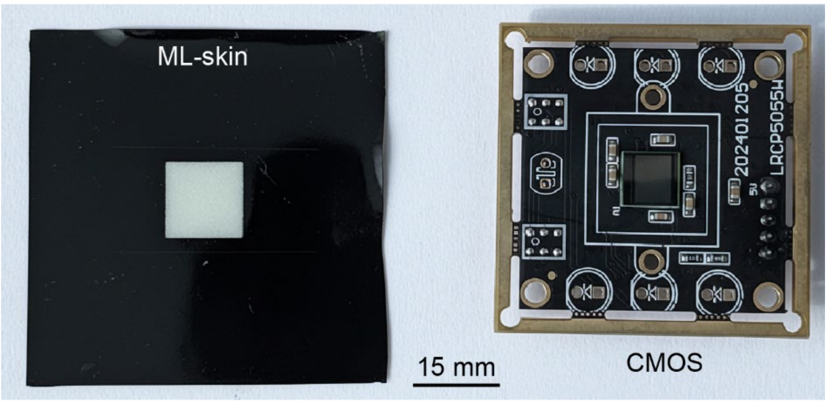

Figure S5. Photographs of the fabricated ML-skin and the CMOS module used for assembling the ML-sensor.

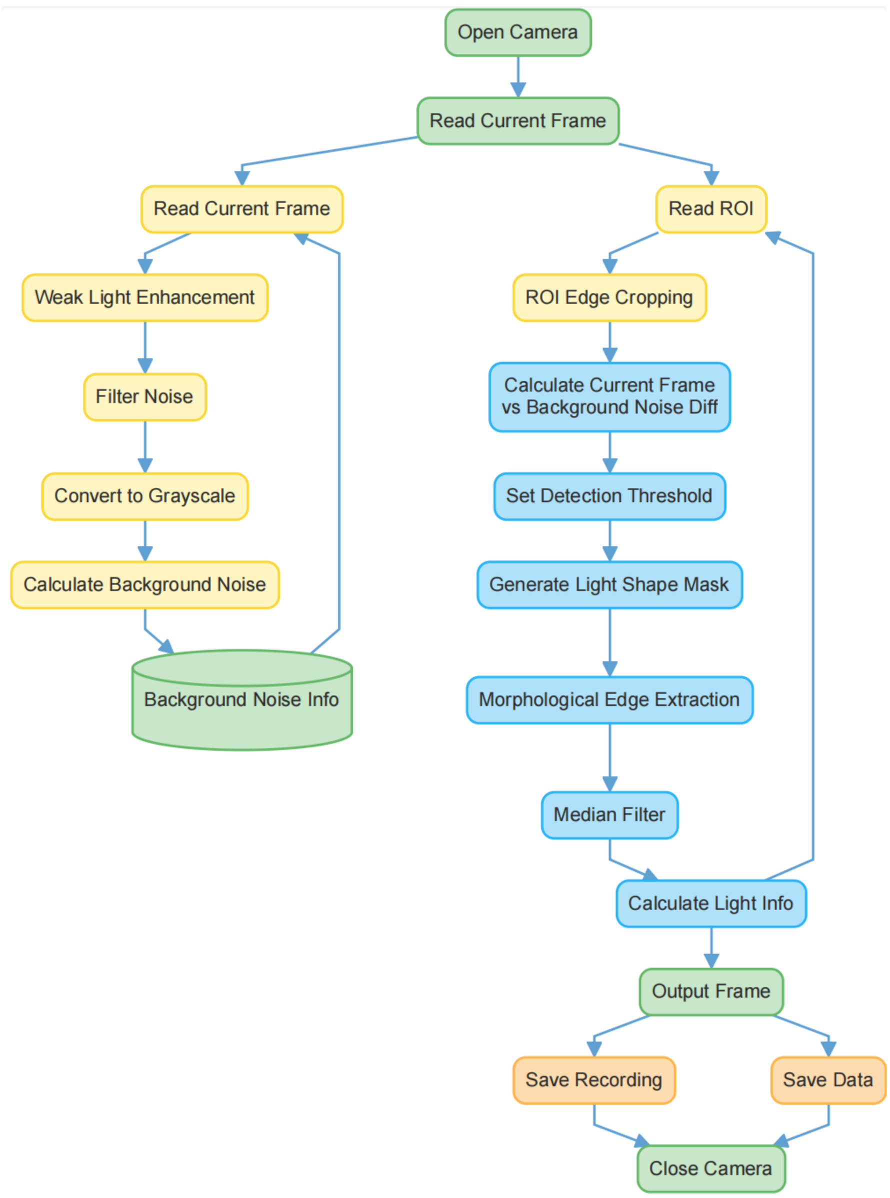

Figure S6. Flowchart of the mechanoluminescent signal processing algorithm used for ML-sensor data analysis.

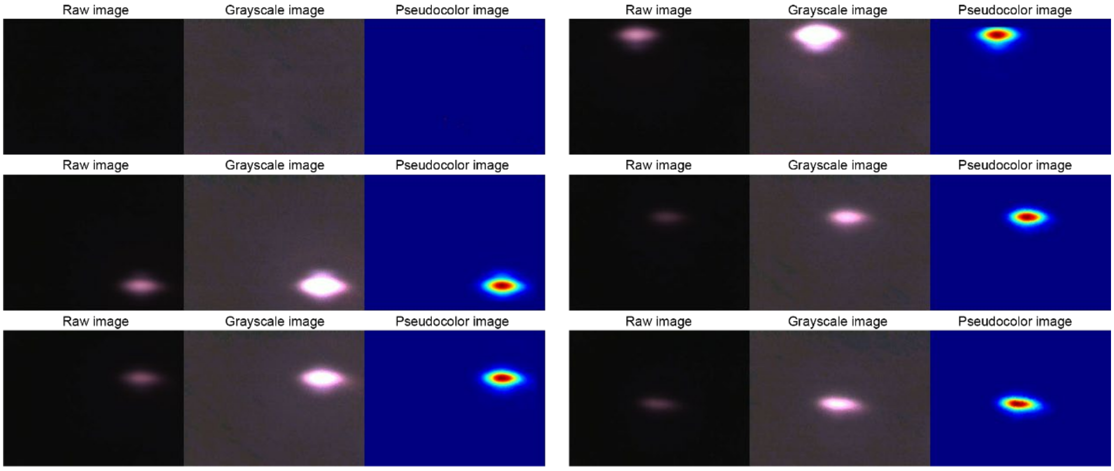

Figure S7. Raw images, grayscale images, and pseudocolor representations of mechanoluminescent signals captured by the ML-sensor.

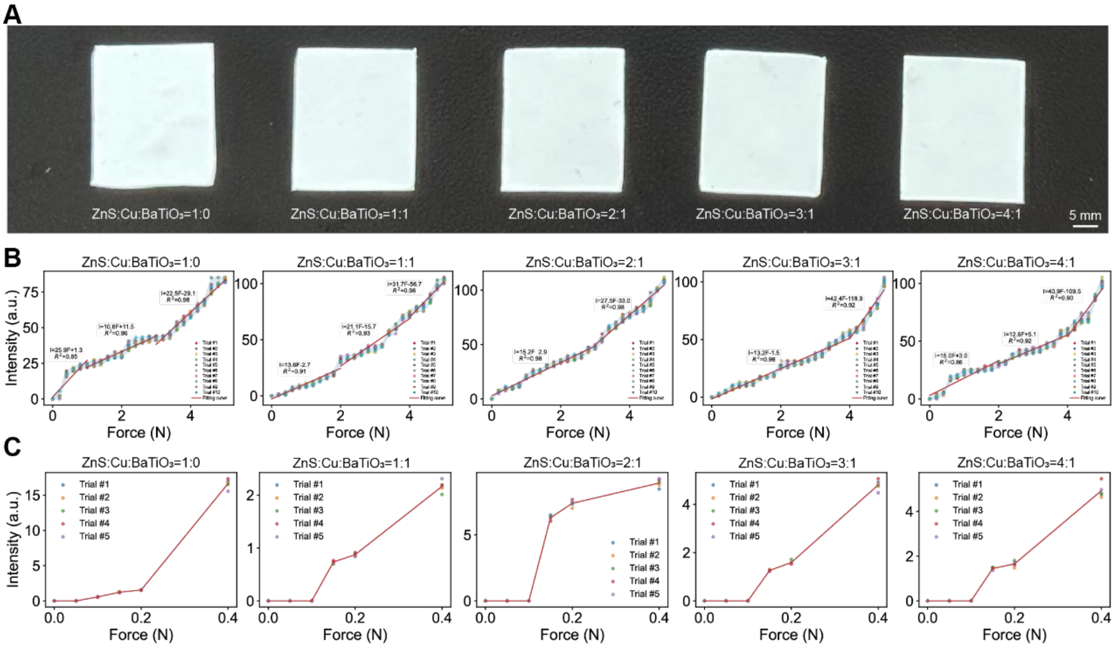

Figure S8. Performance comparison of ML-skins with different ZnS:Cu/$BaTiO_3$ formulations. A) Photographs of ML-skins with different ZnS:Cu/$BaTiO_3$ formulations, with ZnS:Cu:$BaTiO_3$ ratios of 1:0, 1:1, 2:1, 3:1, and 4:1. B) Force-intensity characteristics of ML-skins with different formulations. C) Minimum detectable force of ML-skins with different formulations.

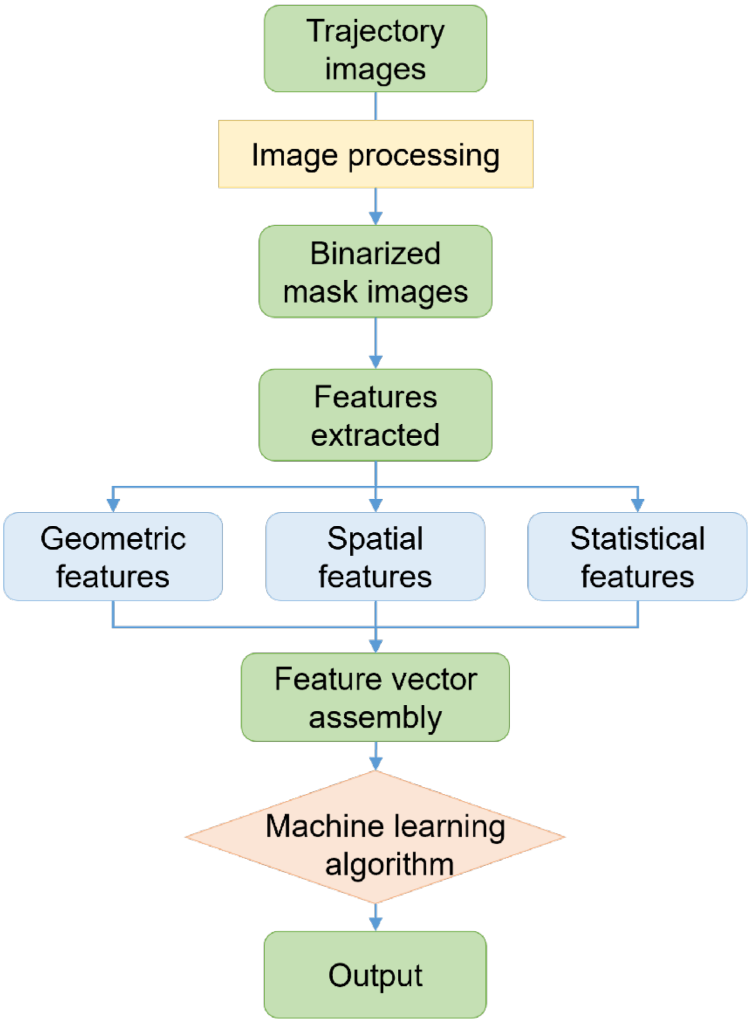

Figure S9. schematic diagram of the process of recognizing the trajectory of handwriting.

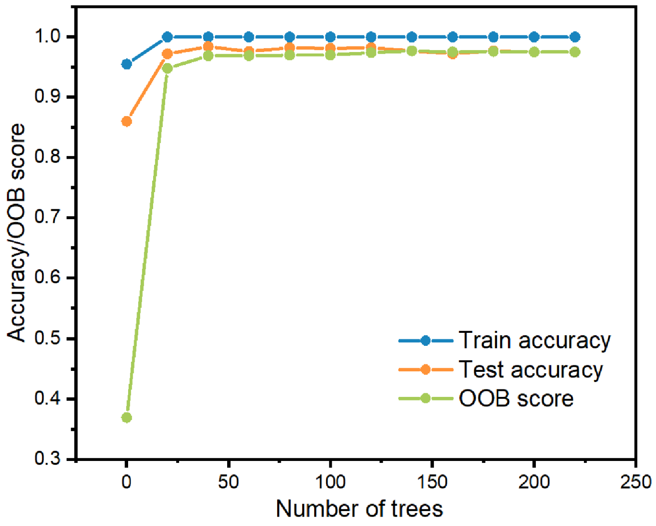

Figure S10. The curves of train accuracy, test accuracy, and out-of-bag score as a function of the number of trees for handwriting recognition.

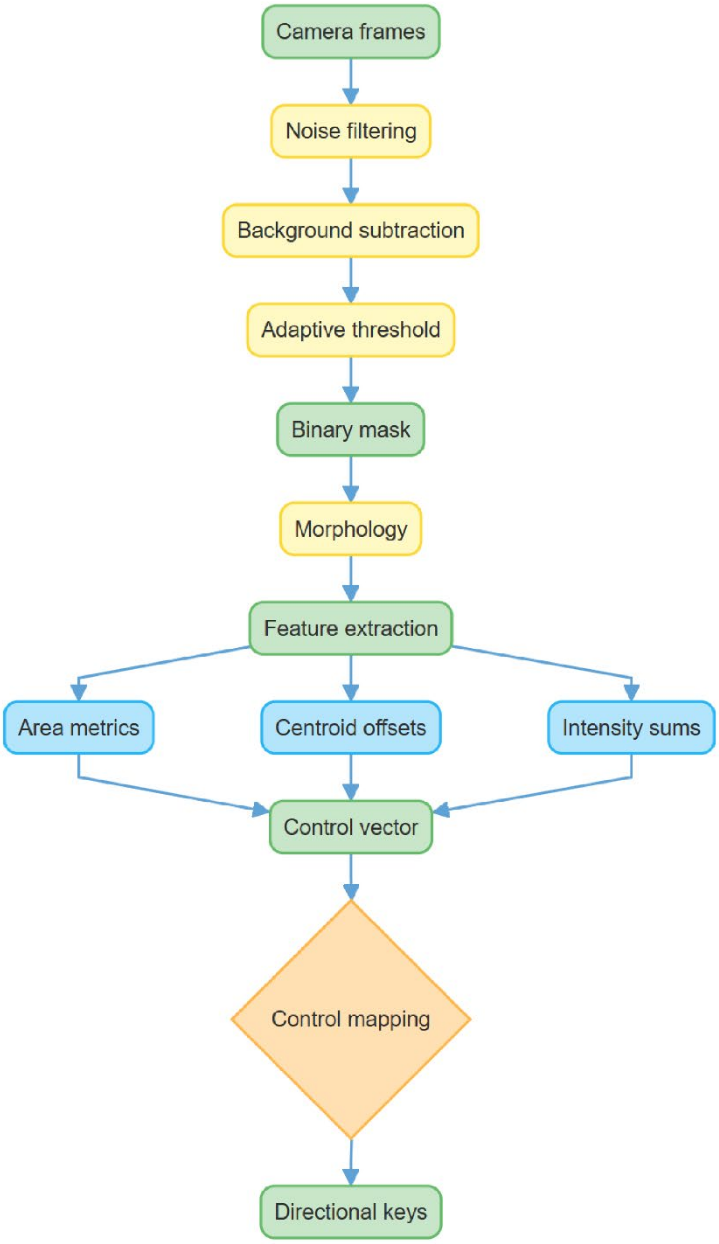

Figure S11. Workflow of the human-computer interaction algorithm based on mechanoluminescent signal features.

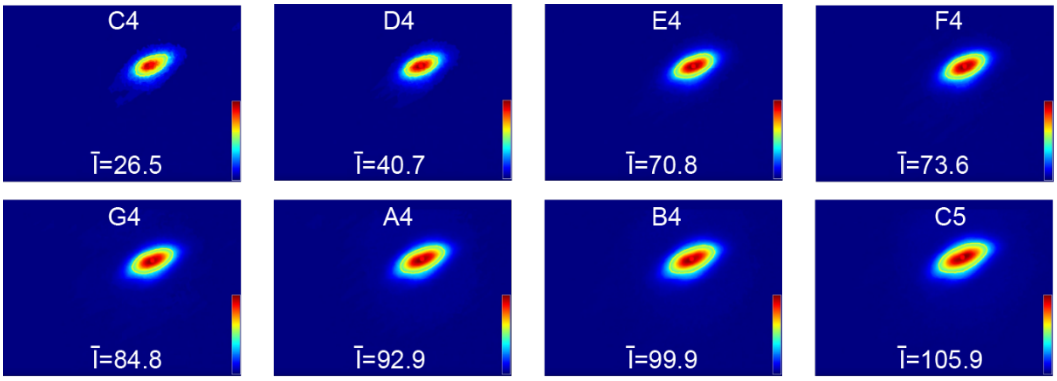

Figure S12. Pseudocolor images while performing a virtual flute via ML-skin.

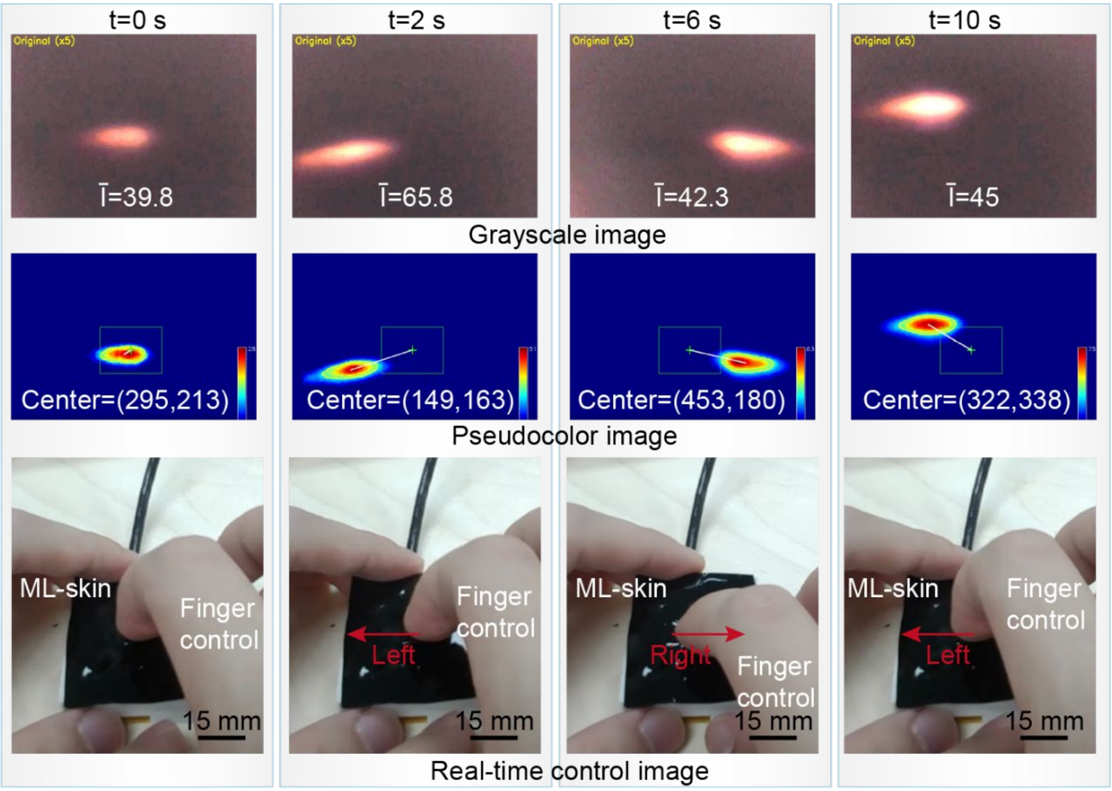

Figure S13. Workflow of the human-computer interaction algorithm based on mechanoluminescent signal features.

Supplementary references

[1] J. Liu, H. Liu, H. Guo, L. Huang, T. Lu, *Adv Funct Materials* **2024**, 2412377.

[2] Z. Ye, S. Fang, T. Zhang, H. Cheng, J. Ou, J. Yu, Y. Zhuang, R. Xie, L. Wang, *Advanced Materials* **2025**, e14909.

[3] J. Yin, Z. Cao, Y. Zhou, X. Huo, Z. Wu, *Small* **2025**, *21*, 2410190.

[4] M. H. Rosle, Z. Wang, M. N. I. Shiblee, K. Ahmed, H. Furukawa, S. Hirai, *IEEE Sensors Letters* **2022**, *6*, 1.

[5] J. Yu, X. Hou, M. Cui, S. Zhang, J. He, W. Geng, J. Mu, X. Chou, *Nano Energy* **2019**, *64*, 103923.

[6] L. Shan, H. Zeng, Y. Liu, X. Zhang, E. Li, R. Yu, Y. Hu, T. Guo, H. Chen, *Nano Lett.* **2022**, *22*, 7275.

[7] X. Yang, W. Yang, W. Wang, K. Li, Y. Li, Q. Zhang, H. Wang, C. Hou, *Device* **2024**, *2*, DOI 10.1016/j.device.2024.100600.

[8] Y. Wan, Z. Qiu, Y. Hong, Y. Wang, J. Zhang, Q. Liu, Z. Wu, C. F. Guo, *Advanced Electronic Materials* **2018**, *4*, 1700586.

[9] M. Kielar, T. Hamid, L. Wu, F. Windels, P. Sah, A. K. Pandey, *ACS Appl. Mater. Interfaces* **2019**, *11*, 21775.

[10] V. K. Chandra, B. P. Chandra, P. Jha, *Appl. Phys. Lett.* **2013**, *102*, 241105.

[11] B. Chen, X. Zhang, F. Wang, *Acc. Mater. Res.* **2021**, *2*, 364.

[12] Z. Huang, X. Li, T. Liang, B. Ren, X. Zhang, Y. Zheng, Q. Zhang, Z. Fang, M. Wu, M. Zulfiqar, L. Jing, S. Qu, B. Chen, J. Gan, D. Peng, *Responsive Materials* **2024**, *2*, e20240019.

[13] A. Qasem, P. Xiong, Z. Ma, M. Peng, Z. Yang, *Laser & Photonics Reviews* **2021**, *15*, 2100276.

[14] C. Deng, Y. Zhang, D. Yang, H. Zhang, M. Zhu, *Advanced Sensor Research* **2024**, *3*, 2300168.

[15] M. Acosta, N. Novak, V. Rojas, S. Patel, R. Vaish, J. Koruza, G. A. Rossetti, J. Rödel, *Applied Physics Reviews* **2017**, *4*, 041305.

[16] M. Dai, H. Chen, F. Wang, Y. Hu, S. Wei, J. Zhang, Z. Wang, T. Zhai, P. Hu, *ACS*

Nano **2019**, *13*, 7291.

[17] Y. Jiang, L. Jin, Y. Huo, *Journal of the Mechanics and Physics of Solids* **2021**, *156*, 104615.

[18] L. Bokobza, *Polymers* **2023**, *15*, 2900.
